# Degradation of Mechanical Properties of Flax/PLA Composites in Hygrothermal Aging Conditions

**DOI:** 10.3390/polym16040528

**Published:** 2024-02-15

**Authors:** Liujiao Wang, Juana Abenojar, Miguel A. Martínez, Carlos Santiuste

**Affiliations:** 1 Continuum Mechanics and Structural Analysis Department, Carlos III University of Madrid, Av. Universidad, 30, 28911 Leganes, Spain; csantius@ing.uc3m.es; 2 Science and Engineering Materials Department, Carlos III University of Madrid, Av. Universidad, 30, 28911 Leganes, Spain; abenojar@ing.uc3m.es (J.A.); mamc@ing.uc3m.es (M.A.M.); 3 Mechanical Engineering Department, Pontificia Comillas University of Madrid, C/Alberto Aguilera, 25, 28015 Madrid, Spain

**Keywords:** biocomposite, hygrothermal aging test, plasma treatment, mechanical properties

## Abstract

The main advantage of green composites is their biodegradability, but this biodegradability can also be considered a drawback if the degradation appears during the service life of the component. Therefore, the study of the mechanical behavior of green composites after hygrothermal aging tests is necessary to analyze their degradation process. This study aims to comprehensively analyze the hygrothermal aging behavior and aging mechanism of flax-fiber-reinforced polylactic acid (PLA) biocomposites. The fully biodegradable composites are manufactured by compression molding. In addition, the influence of atmospheric-pressure plasma treatment on the mechanical properties of the biocomposite is studied. Specimens are exposed to water vapor and 40 °C environmental conditions in a stove for up to 42 days. Several specimens of each type are taken out at regular intervals and tested to examine the water absorption, mechanical properties, and thermal characterization. The results show that the stiffness was significantly reduced after 24 h due to matrix degradation, while the strength was reduced only after three weeks.

## 1. Introduction

The importance of green composites in industry has increased with public environmental awareness. They are prominent in automotive manufacturing components, crashworthiness elements, and orthopedic prostheses [1,2]. In addition, natural fibers are better in thermal and acoustic isolations than synthetic fibers such as glass and carbon fibers. They can potentially be applied in critical applications involving tensile and cyclic loading.

Despite the promising applications of green composites in industry, they face several drawbacks due to their natural features, such as poor fire resistance, high moisture absorption, and lower durability. For instance, the hydrophilic nature of natural fibers causes them to absorb water in a humid environment and swell until saturation, which breaks the bond between the fiber and matrix, leading to delamination and loss of mechanical properties. The accelerated degradation properties of biodegradable plastic matrices (basically those from bio-based polymers) in humid and warm environments also significantly affect the aging results of composites, as they can crystallize, embrittle, split, or be consumed by natural bacteria, leading to material loss [3]. Therefore, the biocomposite is expected to experience accelerated degradation and premature failure under extreme environmental conditions. Many researchers have studied the durability of natural-fiber-reinforced biocomposites under hygrothermal conditions. For instance, Cheng et al. [4] stated in their work that a flax- and carbon-fiber-reinforced PP hybrid composite subjected to water immersion aging at 60 °C for 289 h (~12 days) experienced cracks inside the fibers and the delamination phenomenon between the carbon and flax fibers due to hygrothermal exposure. Moreover, the aging test reduced the mechanical properties of the flax fibers. Fragassa et al. [5] performed a comparative analysis of two flax-fiber-reinforced vinyl ester composite samples. One was dry as a reference, and the other was submerged in saltwater at 80 °C for around 42 days. The micrographic examination of the samples verified that the vinyl ester matrix became brittle with aging. The flexural strength of the composite decreased significantly, and the flax/vinyl ester composite was sensitive to water absorption, causing fiber/matrix interface degradation, matrix cracking, and delamination.

Zuccarello et al. [6] studied the effects of extreme environmental conditions on the tensile strength and interlaminar shear strength (ILSS) of differently orientated laminates made of partial-biobased epoxy and long sisal fiber. They observed that the mechanical behavior of the composite progressively degrades under UV radiation cycles and hygrothermal conditions for up to 8 weeks in terms of both tensile and shear strength. In addition, by comparing the aging-treated composite with a neat matrix after aging, they stated that the sisal fiber does not encourage the degradation of the composite under the accelerated aging test. Furthermore, extreme aging conditions strongly influence the adhesion between the fiber and matrix, and they stated that fiber surface treatment can avoid interfacial debonding.

On the other hand, it is well known that thermoplastic polymer composites have different thermal, chemical, and mechanical behaviors than thermosetting ones. Abderrazak et al. [7] compared a thermoplastic-based matrix composite (flax/Acrylic) with a thermoset-based matrix composite (flax/epoxy). Both underwent a water aging test at room temperature for 30 days. From their results, it can be observed that the tensile characteristics (modulus, strength, and failure strain) of both composite materials generally exhibit a similar evolution under water aging. In hygrothermal conditions, not only does the natural fiber suffer striking consequences of moisture uptake, but also the polymeric matrix, such as PLA, faces severe polymer degradation by hydrolysis, resulting in worse mechanical characteristics of the composite, as reported by [8,9].

For accelerated aging studies on natural-fiber-reinforced composites, the reinforcement could be chemically treated by alkaline, benzoylation, acetylation, and others [10]. The alternative to chemical treatment is the use of physical approaches on the natural fiber surface, such as cold plasma, stretching, and UV radiation [11].

Regarding plasma treatment, it removes silicon and other impurities on the fiber surface. In addition, it also creates polar groups that increase the wettability of the fibers. Then, the treated specimen shows higher moisture absorption due to cavity formation on the material. Moreover, the aging process also increases the wettability of the fibers. The main advantage of plasma treatment with respect to the chemical method is that it does not produce any chemical residue and the environmentally friendly practices are very important in the manufacturing of green composites. However, researchers pointed out that intense plasma exposure of the fiber can cause structural damage and reduce axial strength. After the aging test, they verified that it causes lignin content loss and photo-oxidation and it destroys the adhesion between components. Therefore, its effect on the strength and stiffness of the composite is proportional to the exposure time. Generally, researchers consider that surface treatment on the fibers can effectively enhance the interfacial interaction at the fiber–polymer interface, leading to better force transfer [12].

Enciso et al. [13] studied the effect of high-temperature and hygrothermal conditions on the mechanical weakening of two composites: short coconut-fiber-reinforced LDPE (Low-Density Poly-Ethylene) and short flax-fiber-reinforced LDPE. In addition, natural short fibers were surface-treated with low-pressure plasma (LPP) and compared with untreated ones. From the moisture absorption results and SEM micrographs, they found that the LPP treatment enhances the adhesion between the matrix and reinforcement and builds a barrier against moisture. Aging results demonstrated that high-temperature aging does not significantly influence the mechanical properties of both composites. On the contrary, hygrothermal aging reduces the bending strength and Young’s modulus of both composites. Therefore, they concluded that the mechanical properties of the natural-fiber-reinforced composite are more influenced by moisture than high temperature. In another paper of the same authors [14], they analyzed two composites reinforced with flax fabric. They studied the durability and mechanical properties of these composites subjected to accelerated aging tests and focused on the influence of the Atmospheric-Pressure Plasma Torch (APPT) treatment on the adhesion between the matrix and fiber. They pointed out that the polybutylene succinate (PBS) composite is less durable than the low-density green poly-ethylene (GPE) due to the biodegradability feature of the polyester family under hygrothermal environments. Regarding the APPT fiber surface treatment, they determined that it improves the interfacial bonding between the matrix and reinforcement, showing a higher peeling force of the composite. However, it leads to slightly higher moisture absorption when compared with untreated samples, and the effect of plasma treatment on the mechanical properties is less pronounced. On the other hand, according to this and their previous work [13], non-biodegradable polymers such as GPE and LDPE are more durable than biodegradable polymers. In fact, the moisture absorption test showed that GPE and LDPE barely absorb humidity [14].

Arnaud Regazzi et al. [15,16,17] conducted a study of the aging behavior of flax/PLA biocomposites processed by extrusion and injection molding. They used short flax fibers as reinforcement with a maximum weight ratio of 30%; therefore, their conclusions cannot be extended to the woven composites analyzed in the present study. Green composites combining flax fiber fabric as a reinforcement of PLA matrix have been selected because they have shown great performance in terms of energy absorption under low-velocity impact. Rubio et al. [18] demonstrated that flax/PLA biocomposites can absorb more energy than CFRPs while their specific compressive strength after impact was higher. Moreover, Jiao-Wang et al. [19,20] showed that flax/PLA specimens can be a promising solution for energy absorption structures in the automotive industry and for slender beams subjected to buckling loads [21,22]. Nonetheless, they still need further research to prove their feasibility for industrial applications because the behavior of this green composite under aging tests has not been analyzed.

The present study aims to comprehensively analyze the hygrothermal aging behavior of the woven flax-fiber-reinforced PLA biocomposite made from the heat-compression molding procedure, and the effect of fiber surface treatment with plasma on the mechanical behavior of the biocomposite. More specifically, plasma-treated and untreated flax/PLA samples are exposed to water vapor in 40 °C temperature conditions in a laboratory oven for up to 42 days. Several specimens of each type are taken out regularly and tested to examine the water absorption and mechanical properties such as tensile strength, stiffness, and ultimate strain due to the hygrothermal aging process of the biocomposite material. Scanning electron microscopy (SEM) is also performed to study the interfacial adhesion of unaged samples. Additionally, the aging mechanism is examined by conducting infrared spectroscopy and differential scanning calorimetry on the flax/PLA biocomposites to get a better understanding of the degradation process.

## 2. Materials and Methods

Polylactic acid pellets from NaturePlast, Mondeville, France (commercial name: PLI005, density of 1.24 g/cm^3^ according to ISO 1183 [23], melting temperature of 175 °C, heat deflection temperature (HDT) according to method B120 of about 60 °C, Young’s modulus of 3500 MPa, and tensile strength at break of 50 MPa) were used as the matrix of the biocomposite. Basket-weave (Panama weave) flax fibers not chemically treated acquired from local fabric store, Madrid, Spain (mass per unit area of 463.3 g/m^2^ and thickness of 0.94 mm) were used as the reinforcement.

**Figure 1:**
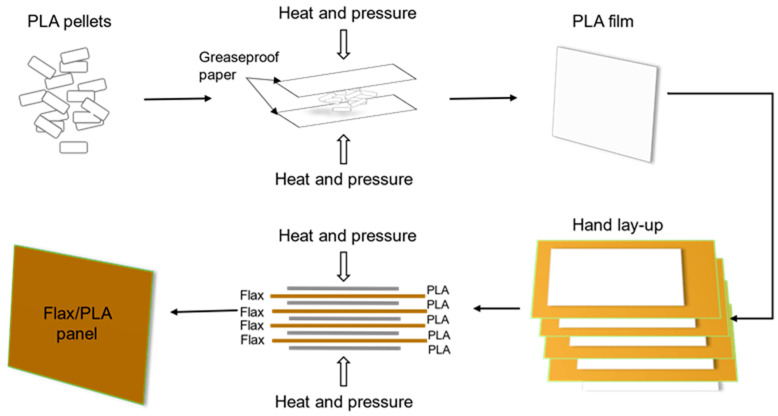
Scheme of flax/PLA plate manufacturing process.

Before manufacturing the biocomposite, the flax fabric and PLA pellets were heated in a 60 °C oven for half an hour to remove moisture. After drying, PLA pellets were melted under 2 MPa of pressure and 180 °C in two heating plates to turn into a thin sheet of 12.3 g and 160 × 200 mm^2^. The fully biodegradable composite laminate was made using hand laid-up process. Five PLA sheets were alternately stacked with four identically orientated flax fabrics and preheated between two heating plates at 180 °C for two minutes. Subsequently, the composite was gradually compressed (maintaining the previous temperature) until reaching the target pressure of 16 MPa. Finally, the flax/PLA plate was removed from the heating plates and naturally cooled down to room temperature. The fiber–weight ratio was 60% (see Figure 1). A more detailed description of the manufacturing process can be found in previous work [22].

**Figure 2:**
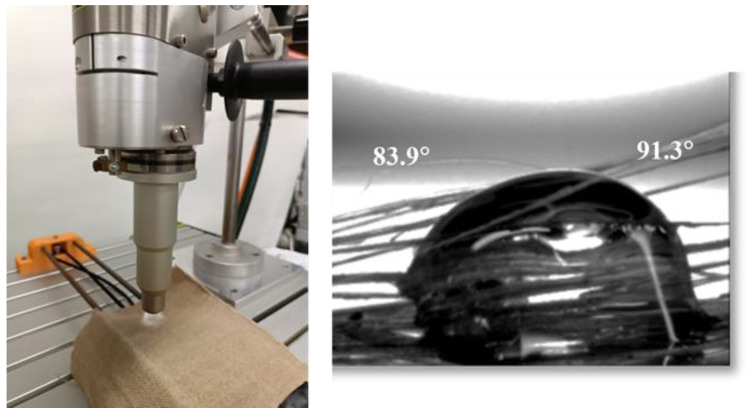
APPT treatment on flax fabric (**left**), PLA pellet on flax fabric at 180 °C (**right**).

For the plasma-treated samples, an additional preparation process was carried out before manufacturing. This treatment was included because of the low wettability of the PLA on flax fibers, as can be observed on the right of Figure 2.

An atmospheric-pressure plasma torch (APPT) device, supplied by Plasma Treat GmbH (Steinhagen, Germany), was used to treat the flax-fiber fabric (Figure 2—left). Potential-free thermodynamic equilibrium plasma treatments (that is, cold) cleaned and activated the fabric surface (composed of mainly polymers), moistening it [24]. This equipment operates at a frequency of 17 kHz and a high-voltage discharge of 20 kV and it is provided with a rotating torch with a ratio of 12.5 mm, ending in a nozzle (1900 rpm) through which the plasma is expelled. The system contains a platform with electronic speed control where the fabric was placed. Air plasma was generated at a working pressure of 2 bar within the rotating nozzle by an off-balance discharge and was expelled through a circular hole onto the samples. The distance between the sample and the nozzle of the plasma torch was set to 10 mm and the speed of the platform was optimized to 41.5 mm/s.

## 3. Experimental Tests

### 3.1. Aging and Tensile Tests

**Figure 3:**
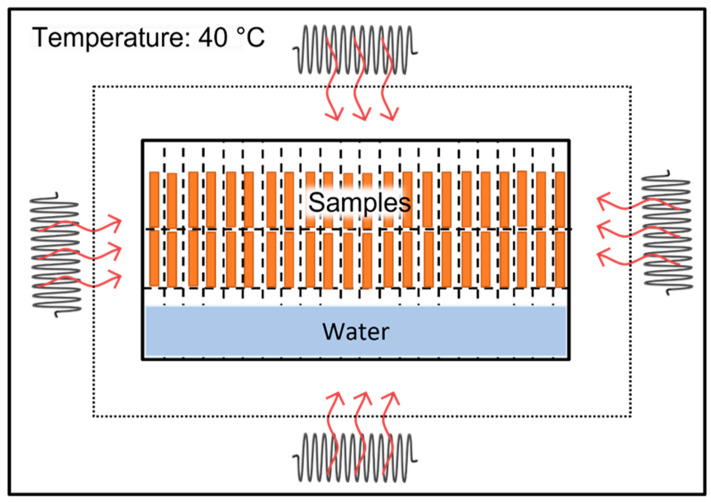
Hygrothermal aging test configuration.

The plasma-treated and untreated flax/PLA composites were cut into 20 × 160 mm^2^ rectangular specimens. The average thickness of flax/PLA specimens before aging was about 2.5 mm. During the aging test, specimens were constantly exposed to hygrothermal conditions in a 40 °C and 100% humidity laboratory oven for up to 42 days (see Figure 3). This aging temperature is higher than the environmental temperature but slightly lower than the glass transition temperature of the PLA polymer to stimulate the polymer degradation [25,26]. Three specimens of each type were taken out at regular intervals to be subjected to different tests to examine the fully biodegradable composite water absorption and mechanical properties, such as tensile strength, stiffness, and ultimate strain due to the hygrothermal aging process.

Uniaxial tensile tests were conducted using a ZwickRoell RetroLine universal testing machine with a cell load of 10 kN, hydraulic grips, and electromechanical actuators. The effective length of the tensile sample was set at 50 mm, and the test speed was established at a constant rate of 1 mm/min. All the uniaxial tensile tests were conducted in the weft direction. The machine recorded force and displacement until final failure. Moreover, infrared spectroscopy and thermal characterization were carried out to analyze the crystallization status of the PLA polymer. Untreated and plasma-treated flax fiber during the aging experiment were compared to study the influence of the plasma treatment.

### 3.2. Contact Angle Experiment and Surface Energy

**Figure 4:**
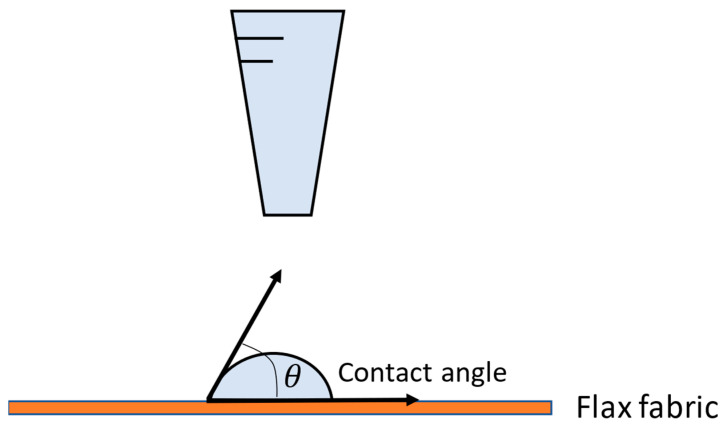
Scheme of wettability test to measure contact angle.

Plasma treatment can increase the hydrophilicity of the fibers, allowing liquids or adhesives to diffuse better or penetrate the fabric. The adhesion between the fiber and the matrix is strongly influenced by surface wettability. Therefore, measuring the droplet contact angle on the fabric surface is essential to study the flax wettability, as shown in Figure 4.

Once the optimized treatment had been carried out, its effect was studied through contact angle measurements. For this, an OCA 15 plus goniometer was used, supplied by Neurtek Instruments (Eibar, Guipúzcoa, Spain). Contact angles were measured by drops of three different liquids: water, glycerol, and diiodomethane. These liquids have different polarities and, therefore, different surface tensions. Once the drop was deposited (as shown in Figure 4), the angles were measured before absorption by capillarity occurred, approximately one minute after the drop was placed. For each condition, five contact angles were measured, representing a total of 10 measurements for each liquid.

The contact angle system OCA 30-2 from DataPhysics Instruments GmbH, Filderstadt, Germany allows the measurement of the angle of the drop on the left and right sides and the surface energy of the system. The equipment can measure a range of 1–180° with an accuracy of ±0.5°. It has a 3× zoom and the software used is the SCA 202 V.3.11.13. In this study, the OWRK method [27] was chosen, which allows for determining the polar component (related to dipole–dipole interactions and hydrogen bonds) and the dispersion (due to London-type forces) of the superficial energy. The polar component is in accordance with the cleaning and surface activation, with the possibility of oxidation on the surface and, therefore, the existence of groups that contain oxygen or nitrogen from the air used as plasma. The treated flax-fiber fabric is used to manufacture the biocomposite within the first hour after treatment.

### 3.3. Infrared Spectroscopy

**Figure 5:**
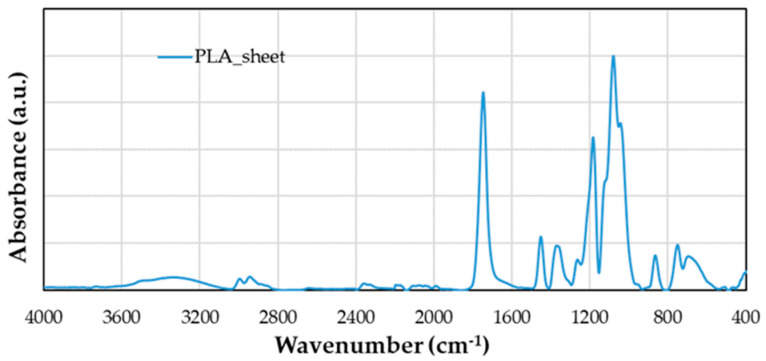
Infrared spectrum of PLA sheet.

Fourier-Transform Infrared Spectroscopy (FTIR) by Brucker Optic GmbH (Madrid, Spain) was used in this work with the attenuated total multiple reflection method (ATR). The produced spectra were approximately at 10 μm in depth and were recorded with a Bruker Tensor 27 spectrometer, which used a diamond prism with a resolution of 4 cm^−1^, 32 scans, and an incident radiation angle of 45°. In addition to recording the spectra of the untreated and plasma-treated flax fiber, PLA pellets, the PLA sheet, and flax/PLA, this technique was used to analyze the water absorbance during the aging process. Each sample was analyzed three times at different points, and the three spectra were compared, even though only one is depicted. One sample was studied for each aging time. Firstly, the infrared spectrum of the pure PLA sheet was obtained as the reference (Figure 5 and Table 1).

The area of each peak or band, in the case of hydroxyls, was measured. At a given time, the value of the area of the corresponding peak was subtracted from that of time zero, divided by the area at time zero and multiplying by 100, and the increase or decrease in percentage was obtained.

(1) $$Area=\left(\frac{{Area}_{tx}-{Area}_{t0}}{{Area}_{t0}}\right)\times 100\%$$

According to the chemical formula of polylactic acid (C_3_H_4_O_2_)n, the observed vibrations are due to C–C, C–H, C=O, C–OH, O–H, and –C–O–C– bonds. Table 1 shows the assignment of the bands and the peak where they take place [28,29].

### 3.4. Thermal Characterization

Differential scanning calorimetry (DSC) analysis was performed on PLA pellets and PLA sheets to compare these thermograms with flax/PLA biocomposites. A DSC 822e supplied by Mettler Toledo GmbH (Greifensee, Switzerland) was used to examine the specimen’s thermal properties and evaluate the aging effect on the biocomposites. The scans were performed with a heating rate of 20 °C/min in a temperature range of −20 °C to 200 °C. Aluminum crucibles of 40 μL were used and filled with 8–10 mg of PLA or 10–15 mg of the composite. The DSC tests were repeated three times for each condition and material to verify the repeatability of the results. At the same time, changes in the crystallinity (X_c_) of PLA and flax/PLA composites were also studied using differential scanning calorimetry (DSC), according to Equation (1):

(2) Xc=∆Hm−∆HcθPLA∆Hm100%PLA×100%

where $∆H_{m}$ is the melting heat, $∆H_{c}$ is the crystallinity peak, $\theta_{PLA}$ is the PLA fraction, and ${∆H_{m}}_{100\%PLA}$ is the melting heat for fully crystalline PLA (93.6 J/g) [30]. In addition, the glass transition temperature (*T_g_*), melting temperature (*T_m_*), and the amount of moisture absorbed by the composite can be calculated from DSC curves.

The aim of this study is the determination of changes resulting from aging, such as water adsorption and alterations in *T_g_*, *T_m_*, and crystallinity. These changes are observable only in the first scan. Performing a subsequent second scan, given the rapid cooling of the thermoplastic (10 or 20 °C/min), prevents the material from crystallizing. As a result, only one *T_g_* is observed, and the processes occurring during aging are unobserved [31].

## 4. Results

### 4.1. Mechanical Response of Hygrothermally Aged Flax/PLA Samples

**Figure 6:**
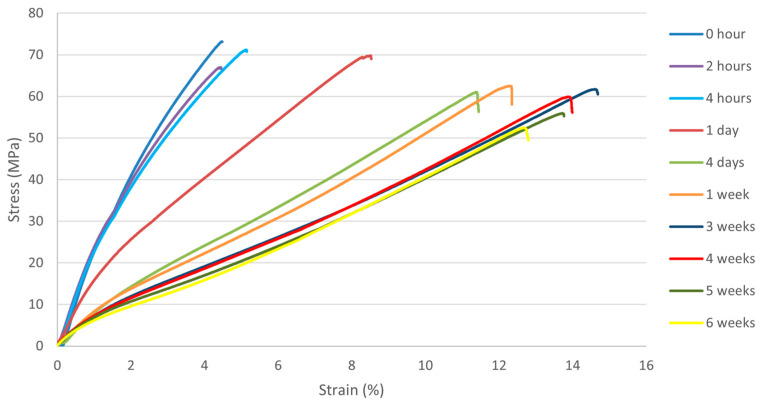
Stress–strain diagram of flax/PLA biocomposite for different aging times.

The stress–strain curves of flax/PLA specimens obtained from the uniaxial tensile tests are shown in Figure 6. As mentioned above, a set of six specimens was extracted from the oven periodically and tested. Only the most representative specimen was chosen to establish a comparative analysis of different aging times in Figure 6. The results show that stiffness decreases significantly with aging time, while ultimate strain increases with aging. In addition, it can be observed that the maximum strength drops from 73.2 MPa to 61.7 MPa within three weeks of aging, which is a significant reduction of at least 14%.

The effect of aging can be observed not only in the stiffness and ultimate strain but also in the shape of the stress–strain curves. The flax/PLA biocomposites have shown nonlinear behavior in previous works, including nonlinear elasticity and viscoplastic behavior [19,32,33]. There is a clear nonlinearity for stress–strain curves of aging time up to 24 h, where the stiffness decreases with strain. However, from four days of aging, the mechanical response of the biocomposite becomes almost linear. Both flax fiber fabrics and PLA polymers have inherently linear-elastic behavior, whereas the composites that combine both components, as studied in previous works, exhibit a nonlinear mechanical response that can be attributed to the matrix–fiber interaction. It is, therefore, reasonable to assume that aging conditions of at least four days are sufficient to break the interaction between fiber and polymer interfaces in the composite, leading to a linear behavior controlled by the flax fiber fabric.

**Figure 7:**
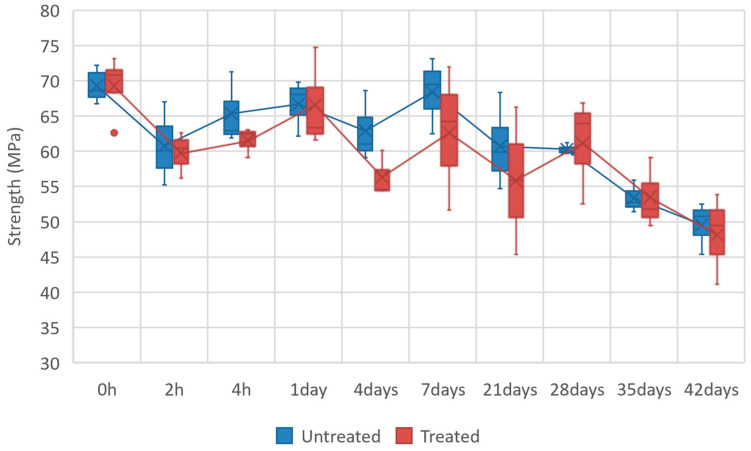
Tensile strength of samples over aging time. Comparison between plasma-treated and untreated samples.

The strength for all the specimens is plotted in Figure 7 as a boxplot to represent the results’ dispersion, a typical phenomenon of natural-fiber-reinforced composites. Firstly, according to the results, both trend lines (untreated and plasma-treated) exhibit a similar tendency despite the striking standard deviation. Notably, the plasma-treated batch of results display a higher data dispersion than the untreated ones, potentially a random phenomenon, and the mean values of treated samples are generally smaller than those of untreated samples. Consequently, the impact of plasma treatment on the tensile strength can be considered negligible.

On the other hand, the highest tensile strength, 70 MPa, was found in the control specimens (starting state). The hygrothermal aging condition considerably influences the tensile response of the biocomposite because the strength drops quickly after only two hours of conditioning. Then, there is a plateau around 65 MPa until seven days of aging. Subsequently, the tensile strength decreases from 21 days until the maximum aging time of 42 days, reaching the lowest strength of around 50 MPa, as shown in Figure 7.

**Figure 8:**
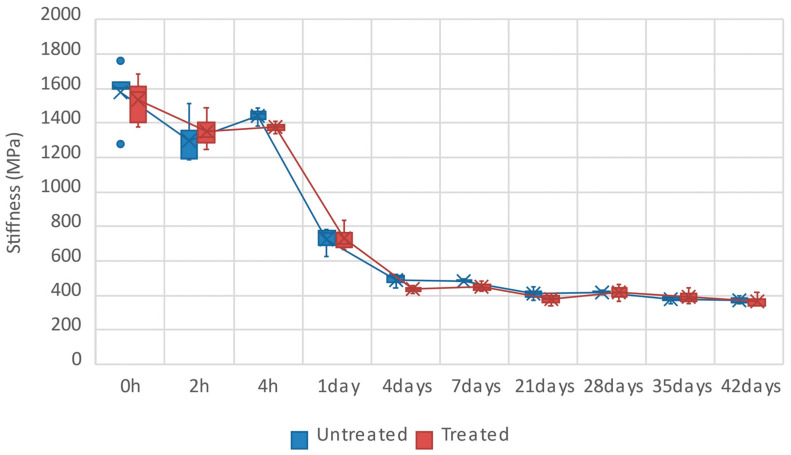
Tensile stiffness of samples over aging time. Comparison between plasma-treated and untreated samples.

Figure 8 compares the stiffness of treated and untreated flax/PLA biocomposites at different aging times. Since the mechanical behavior observed in stress–strain curves is clearly non-linear, the average stiffness plotted in Figure 8 is defined as strength divided by ultimate strain. As in tensile strength, the stiffness of both treated and untreated samples barely shows differences; therefore, plasma treatment does not influence the stiffness. In addition, the dispersion observed in this case is much lower than in the strength. The main reason is that the strength of the flax/PLA biocomposite is more dependent on the quality of the natural fibers and defects during the manufacturing process.

The specimens with an aging time between two and four hours show a slight reduction of 9% and 18% compared to the control specimens (average stiffness around 1600 MPa). After that, the average stiffness at 24 h is drastically reduced between 52% and 54%, and the decline in the stiffness continues until the fourth day of aging, between 69% and 72%. Lastly, average stiffness gradually stabilizes and remains at 400 MPa for aging times higher than four days.

In summary, the effect of hygrothermal aging on the mechanical behavior of flax/PLA composites is the drastic reduction in average stiffness in 24 h, including a loss of nonlinearity of the stress–strain curve. However, the reduction in strength is observed for aging times higher than 21 days. This behavior can be attributed to different times of degradation for the matrix and fibers. First, matrix degradation starts after 24 h, leading to a poor fiber–matrix adhesion that implies a stiffness reduction and loss of curvature in stress–strain diagram. Second, the fiber degradation after three weeks leads to a strength reduction. Therefore, to verify these hypotheses, more studies on the effect of hygrothermal aging were conducted, which include moisture absorption, IR spectroscopy, and thermal characterization.

**Figure 9:**
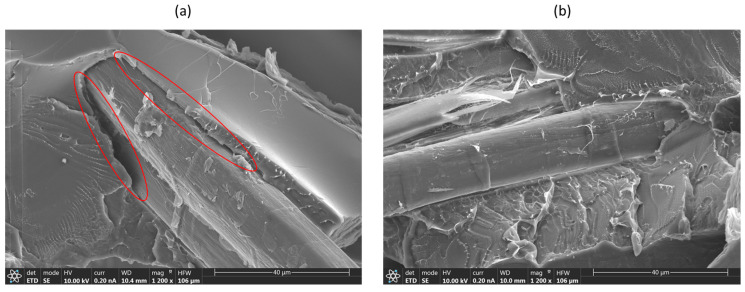
SEM micrographs of unaged flax/PLA after tensile test: (**a**) fracture section of the untreated sample, red circles highlight the low fiber–matrix adhesion of the biocomposite; (**b**) the fracture section of the plasma-treated sample.

Nevertheless, although there is only a slight difference in mechanical properties (strength and stiffness) between plasma-treated and untreated flax/PLA composites, a significant contrast is observed in the scanning electron microscopy (SEM) of the fracture section for unaged samples, as illustrated in Figure 9.

In Figure 9b, the plasma-treated sample exhibits enhanced adhesion between the fiber–matrix interfaces, indicating that the APPT treatment on the fabric’s surface has notably improved the adhesion of the PLA polymer with the flax fiber. In contrast, Figure 9a shows the untreated sample with evident separation along the fiber direction.

### 4.2. Moisture Absorption

**Figure 10:**
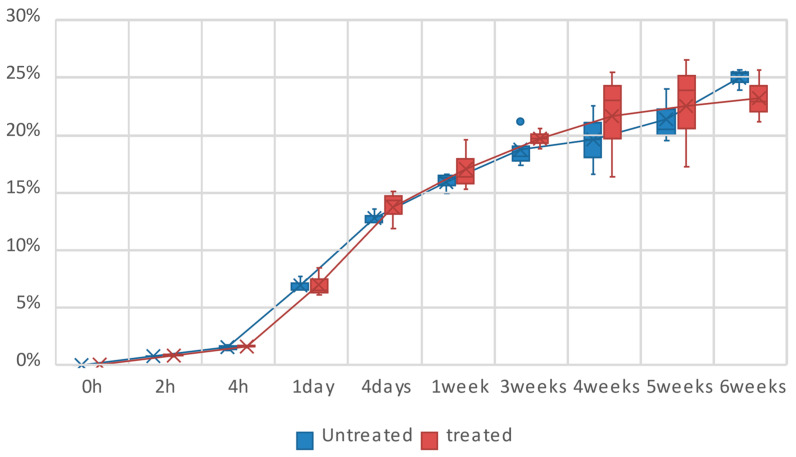
The average weight variation over time. Comparison between plasma-treated and untreated samples.

Figure 10 shows the weight variation as a function of aging time for untreated and plasma-treated flax/PLA specimens. It aims to analyze the moisture absorption of biocomposites subjected to high-humidity environments. The results show that, even after six weeks of aging, the specimens are not saturated and they continue to absorb water, although at a much slower rate than during the first week of the experiment.

On the other hand, as in the case of strength and stiffness, the difference between treated and untreated specimens is lower than the standard deviation. Firstly, the difference between both results is 4.24%, 5.08%, and 0.04% for the first 2 h, 4 h, and 24 h, respectively. Later, this difference increases, but the maximum variation between treated and untreated samples is only 9.53% after four weeks of aging, and the plasma-treated samples show higher weights than the untreated ones. Therefore, these differences can be considered negligible.

### 4.3. Contact Angle

The measured contact angles are shown in Table 2, and it can be observed that the contact angles of untreated fibers are greater than 90°, but the diiodomethane has a contact angle close to 90° because it is a dispersive liquid. However, the contact angles of treated fibers are much lower than 90° for the three liquids. Therefore, plasma treatment has a clear effect on the reduction in contact angle.

On the other hand, the surface energy of flax fibers can be obtained through the contact angle experiment. Table 3 shows that the total surface energy has increased by 68% for those plasma-treated samples, and the polar surface energy has increased by 90% due to the favorable wettability with polar liquids such as water and glycerol. Therefore, the higher the surface energy, the better the liquids will spread out on the fiber surface. It is verified that the plasma can effectively enhance the wettability of flax fibers.

### 4.4. Infrared Spectrum

**Figure 11:**
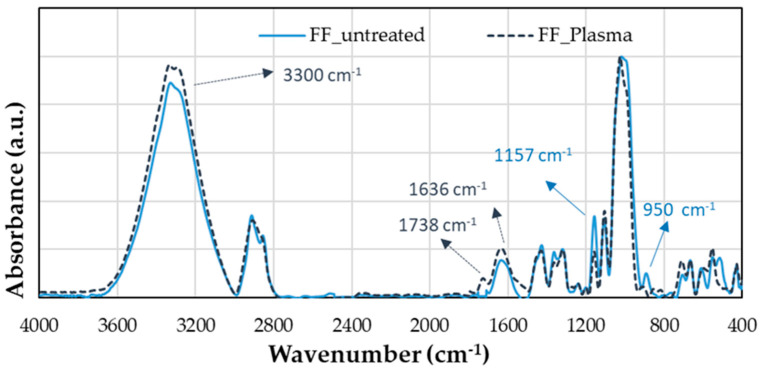
Infrared spectrum of flax fibers before and after plasma treatment.

Figure 11 shows the infrared spectrum of flax fibers before and after plasma treatment, and the assignation of peaks was carried out according to Table 1. In the range between 3000 and 3600 cm^−1^, there is a peak at 3300 cm^−1^ related to the stretching vibration of the hydroxyl group (OH) from the cellulose and lignin structure. In addition, this peak increases with the plasma treatment. The absorption band (doublet) in the range of 3000–2800 cm^−1^ corresponds to C–H of CH_2_ asymmetric and symmetric stretching, which does not show significant changes after plasma treatment. However, according to Fiore et al., this stretching vibration can be produced by CH and CH_2_ in cellulose and hemicellulose [34]. The central region from 2800 to 1800 cm^−1^ does not present any significant change.

Nevertheless, at 1738 cm^−1^ a small peak appears in the plasma-treated curve, corresponding to the carbonyl group (C=O). At 1636 cm^−1^, there is a second peak due to the deformation vibration of OH, and it could be associated with the absorbed water in the crystalline cellulose of flax fibers treated with plasma [35]. Therefore, it shows that an increase in the polar groups of the fiber is occurring, leading to an oxidation process in the material [36]. This increase agrees with the increment in surface energy observed in Table 3.

On the other hand, the infrared spectrum of plasma-treated fiber presents two peaks lower than the untreated fiber. The first peak is at 1157 cm^−1^ due to the stretching vibration of C–O–C, and the second is at 950 cm^−1^ due to the deformation vibration of C–H. These peaks may be oxidized to form C=O and more OH groups. Finally, the most remarkable peak from the infrared spectroscopy is the deformation vibration of C–OH at 1022 cm^−1^, in which the spectrum after plasma treatment is narrower than before [29].

**Figure 12:**
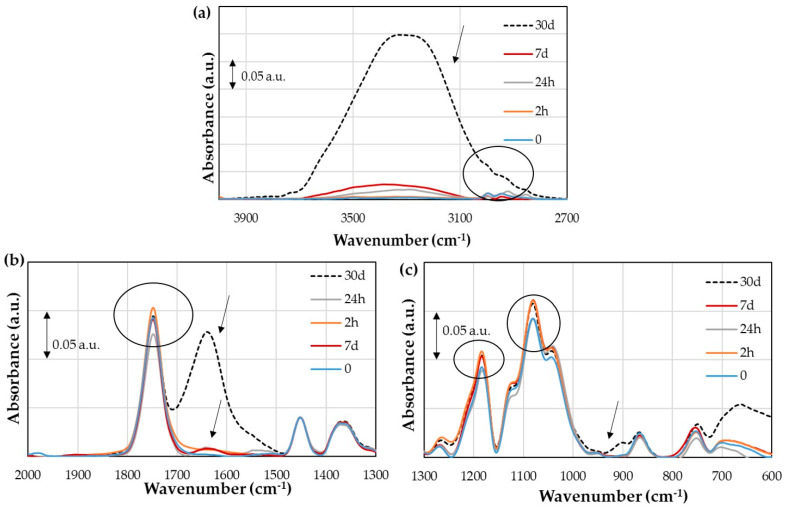
Infrared spectrum of untreated flax/PLA biocomposite: (**a**) wavenumber region from 4000 to 2700 cm^−1^; (**b**) wavenumber region from 2000 to 1300 cm^−1^; (**c**) wavenumber region from 1300 to 600 cm^−1^.

The IR spectrum of the untreated flax/PLA biocomposite has the same peaks as that of the PLA sheet (Figure 5). Figure 12a shows the IR spectrum of the untreated flax/PLA biocomposite with the region between 4000 and 2700 cm^−1^ corresponding to OH and CH_3_ stretching. The IR spectroscopies for aging times lower than 30 days significantly differ from 30 days. Then, it is difficult to clearly distinguish the peaks of the stretching vibrations of CH_3_ at each aging stage. However, from the general trend, as the aging time increases, the moisture absorption increases. The area of each region is 34%, 799%, 1506%, and 19771% at 2 h, 24 h, 7 days, and 30 days, respectively.

From Figure 12b, the carbonyl groups (C=O) at 7 and 30 days at 1748 cm^−1^ coincide with unaged specimens. At 2 h, the absorbance is slightly higher than those of the others, and at 24 h, it is the lowest. This phenomenon may be due to some rearrangement of C–O bonds when the composite absorbs moisture. Moisture absorption is confirmed by the appearance of the deformation vibration of OH at 1636 cm^−1^, which begins to grow at 7 days as a small peak and reaches the same absorbance level as the carbonyl peak at 30 days.

Finally, it can be seen from Figure 12c that the absorbances at 2 h, 7 days, and 30 days are higher than those of unaged specimens at 1182 cm^−1^ (C–O–) and 1077 cm^−1^ (C–O–C), while the aging time at 24 h is the same as those of unaged specimens. In addition, a small peak starts to form around 900 cm^−1^, related to the O–H out-of-plane deformation vibration.

**Figure 13:**
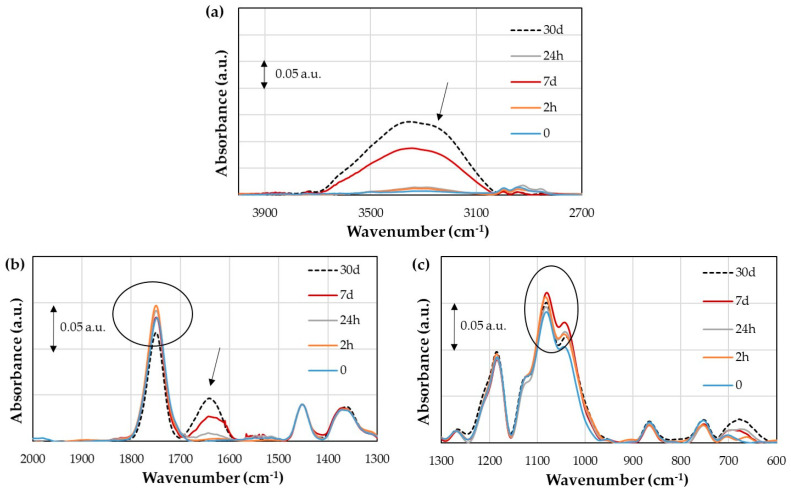
Infrared spectrum of plasma-treated flax/PLA biocomposite: (**a**) region from 4000 to 2700 cm^−1^; (**b**) region from 2000 to 1300 cm^−1^; and (**c**) region from 1300 to 600 cm^−1^.

Figure 13a points out that the plasma treatment reduces the moisture absorption of flax fibers for long aging periods compared to untreated flax fibers; i.e., at 30 days, the area under the region of the treated fiber is 3236%, while it is 19771% for the untreated one (keeping in mind that the untreated area includes the CH_3_ area). The same phenomenon is observed at 24 h, where this area is also reduced from 779% to 223%. However, the absorption is higher at 2 h (163%) and 7 days (2021%). In addition, a peak at 1636 cm^−1^ can be observed (Figure 13b—marked with an arrow), which corresponds to the deformation vibration of OH. Minor differences also appear for the carbonyl groups at 1749 cm^−1^. An increment is observed at 2 and 24 h, and at 30 days, a slight decrement is observed (Figure 13b—marked with a circle). Figure 13c shows the region from 1300 to 600 cm^−1^. The differences between aging times are similar than in the IR spectrum of the untreated biocomposite at 1080 cm^−1^ (C–O–C). This peak has an increment at 2 h by 15% and 12% for untreated and treated fibers. At 4 h, it decreases until 2% and 4%, it grows back at 7 days by 15%, and then it decreases until 13% and 8% at 30 days. These small changes may agree with hydrolysis or absorption–desorption processes on a very small scale and are reversible.

In general, slight differences were found for vibrations C=O, C–O, and C–O–C, both for untreated and treated fiber composites. They do not have a fixed sequence, so it may be a consequence of the spectrum itself. However, since the working temperature is lower than the PLA glass transition temperature, no acid medium was observed, and the medium was two units more basic, the PLA hydrolysis process could not have occurred. In addition, no crystallinity change is expected, since this would be reflected in the carbonyl peak (C=O) and shift towards higher wavenumbers. The shift would indicate a change in the polymer structure, specifically the PLA crystallization [37].

Therefore, even when the wettability of flax fibers was considerably increased by the plasma treatment, the effect on the mechanical properties was negligible because the plasma treatment was not able to modify the bonds necessary to improve the fiber–matrix compatibility.

### 4.5. Thermal Characterization

**Figure 14:**
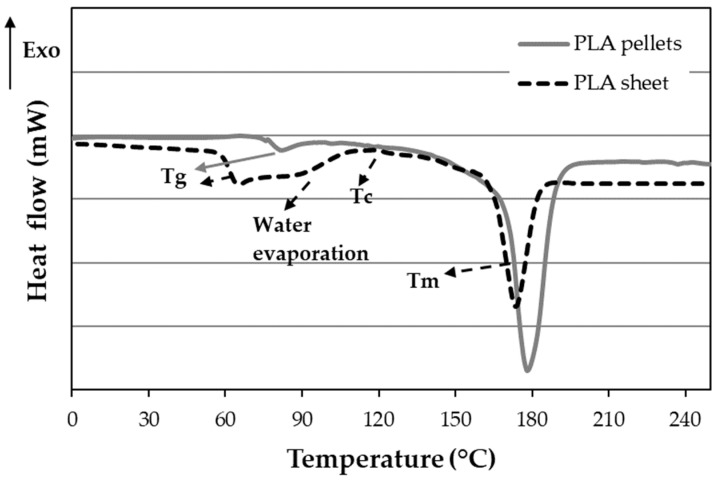
DSC of PLA pellets and sheet.

The PLA pellets have a *T_g_* of 79 ± 1 °C and a melting temperature of 178 ± 1 °C with an enthalpy of 52 ± 2 J/g (Figure 14). This enthalpy equals 56% crystallinity, not observing a recrystallization peak. However, when PLA pellets are heated to turn into PLA sheets to manufacture the biocomposites, the polymer suffers changes. The *T_g_* decreases to 62 ± 2 °C, followed by a small crystallization peak at 108 °C of 5 J/g (Figure 14). The melting peak also occurs below 173 ± 1 °C with an enthalpy of 29 ± 2 J/g. The crystallinity of the sheets decreases considerably to 26%. The crystallinity of 26% will be the reference for the biocomposites. According to *T_g_* and *T_m_*, the stereochemical conformation of PLA used in this work is Poly (L-lactide) or Poly (D-Lactide) isotactic (LLLLLL or DDDDDD) [38].

**Figure 15:**
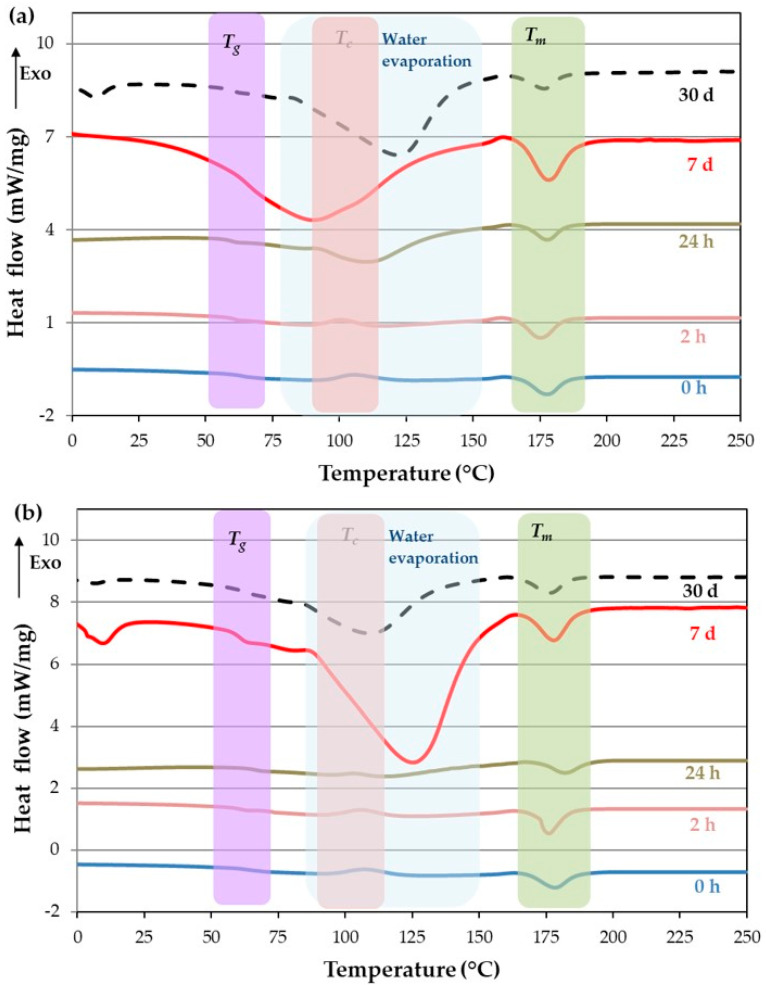
Study of aging evolution for composites flax/PLA: (**a**) untreated flax fiber and (**b**) plasma-treated flax fiber.

Compared to pure PLA, the biocomposites have a lower *T_g_*, from 79 °C for PLA to 63 °C for biocomposites before aging (Table 4). The *T_g_* tends to decrease during aging, although the set of all of them would be 60 ± 3 °C, and they would statistically correspond to the same data group. The enthalpy of the recrystallization peak in the biocomposite is initially slightly higher than that of the PLA sheet (Figure 14) at 0 and 2 h, but *T_m_* decreases. From 24 h to 30 days, $∆H_{c}$ is small and difficult to measure since there is an overlap in the zone of water evaporation, as observed in Figure 15.

On the other hand, Tm has a slight variation, and its average value is 178 ± 2 °C (Table 4). However, considerable differences are found in $∆H_{m}$, which is lower than the $∆H_{m}$ of the PLA sheet. Simultaneously, $∆H_{m}$ affects the crystallinity, which is higher than the PLA sheet and presents a significant variability without having a clear tendency.

The increase in and variability of crystallinity may be due to four factors: (1) Flax fibers may have a nucleating effect due to cellulosic fibers, which is more critical for untreated fibers, increasing the crystallization [39,40]. In this case, aging for a long time also increases crystallinity, being more critical for the treated flax fiber at 30 d. (2) The lack of recrystallization or the overlap with the water evaporation peak after 24 h of aging also produces an increase in crystallinity. (3) Although the amount of fiber was estimated at 60 wt.%, deviations from this quantity may occur. (4) The small samples used for the DSC test may contain different percentages of fibers due to the non-uniform distribution of the fiber–weight ratio.

As Figure 15 shows, the water evaporation peak ranges from 70 °C to 140 °C with a peak at 110 °C. For samples aged for 24 h, the water absorption is 48 J/g and 12 J/g for the untreated and treated fiber, respectively. Since aging time increases, water absorption also increases. Thus, the water evaporation peak for untreated fibers increases to 81 and 218 J/g at 7 d and 30 d, respectively. However, if the fibers are treated, water absorption increases to 195 and 94 J/g at 7 d and 30 d, respectively. This result means treated fiber biocomposites saturate sooner than untreated fiber biocomposites, producing a desorption of water.

## 5. Conclusions

The mechanical behavior of flax-fiber-reinforced PLA biocomposites after hygrothermal aging was analyzed and the aging mechanisms were examined. The influence of APPT treatment on the flax fabric surface to improve the interfacial adhesion and wettability of the biocomposite was studied by the comparison of the results of treated and untreated specimens in tensile tests, contact angles, weight absorption, infrared spectroscopy, and differential scanning calorimetry. The main conclusions are summarized as follows:

- The hygrothermal aging condition considerably influences the mechanical behavior of the biocomposite. It produces a drastic reduction in stiffness in the first 24 h and ends up with a maximum of 72% less than the unaged samples in 6 weeks. The biocomposite also suffers a loss of nonlinearity of the stress–strain curve. In addition, a reduction in the material strength of 14% is observed, but only after aging times higher than three weeks.
- The effect of the APPT treatment on the tensile strength and stiffness is negligible because it does not modify the C=O, C–O, and C–O–C bonds, which are necessary to improve the fiber–matrix compatibility. However, plasma treatment has notably improved the adhesion of the PLA polymer with the flax fiber according to the SEM scans.
- On the other hand, plasma treatment does not significantly increase the moisture absorption of the flax/PLA samples under high-humidity conditions. However, it does make the treated fiber biocomposites saturate sooner than untreated fiber biocomposites, producing water desorption. In addition, the wettability of the material is also enhanced.
- The maximum aging time of hygrothermal aging conditions is six weeks. The stiffness degradation is stabilized at this time, but the strength can probably decrease for higher aging times.
- After 42 days of aging tests, the biocomposite deterioration produces a stiffness reduction of 72% and a strength reduction of 31%.

Finally, more test campaigns, such as the double cantilever beam (DCB) and peeling tests, should be carried out to study the degree of adhesion and cohesion behavior between the matrix and fiber reinforcement before and after being aged under high-humidity and -temperature conditions.

## Figures and Tables

**Table 1:** Assignation of peaks in the IR spectrum of PLA sheet.

Wavenumber (cm^−1^)	Vibrations	
745	C–H/C–O	bending
857	C–C	str ^*^
1039	–C–CH_3_	str
1077	C–O–C	asym ^*^ str
1125	C–H	rocking
1182	C–C/–C–O–	str
1363	C–O	str
1447	CH_3_–C–O	bending
1747	C=O	str
2909	C–H (CH_3_)	sym ^*^ str
2981	C–H (CH_3_)	asym str
3071-3600	OH	str

^*^ asym: asymmetric; sym: symmetric: str: stretching

**Table 2:** Contact angle of flax fabrics before and after plasma treatment. Mean value ± standard deviation.

	Liquids	Water	Glycerol	Diiodomethane
Flax Fabric		Contact Angle (°)		
Untreated		136 ± 6	130 ± 3	92 ± 1
Plasma-treated		53 ± 4	61 ± 3	32 ± 3

**Table 3:** Surface energy of flax fabrics before and after plasma treatment. Mean value ± standard deviation.

Flax Fabric	Surface Energy (mN/m)		
	Total	Dispersive	Polar
Untreated	15 ± 1	14 ± 1	1 ± 0
Plasma-treated	47 ± 2	34 ± 2	12 ± 2

**Table 4:** Thermal data obtained by DSC measurements for flax/PLA: aging evolution.

Aging Time	Flax	Composite Flax/PLA					
		*T_g_* (°C)	*T_c_* (°C)	Δ*H_c_* (J/g)	*T_m_* (°C)	Δ*H_m_* (J/g)	X_c_ (%)
0	Untreated	63 ± 2	105 ± 2	6.9 ± 0.5	178 ± 1	19.9 ± 0.5	35 ± 3
	Treated	64 ± 3	107 ± 1	8.1 ± 0.8	178 ± 2	17.2 ± 0.2	24 ± 2
2 h	Untreated	60 ± 1	101 ± 2	6.7 ± 0.2	175 ± 2	22.9 ± 0.8	43 ± 5
	Treated	60 ± 2	106 ± 3	7.2 ± 0.4	176 ± 1	19.0 ± 0.3	31 ± 3
24 h	Untreated	59 ± 1	92 ± 5	1.0 ± 0.6	178 ± 2	15.3 ± 0.6	38 ± 4
	Treated	59 ± 2	100 ± 3	1.8 ± 0.4	182 ± 3	12.1 ± 0.8	28 ± 2
7 d	Untreated	53 ± 2			178 ± 1	16.2 ± 0.7	43 ± 7
	Treated	60 ± 4	87 ± 4	1.5 ± 0.7	178 ± 1	14.7 ± 0.4	35 ± 3
30 d	Untreated	60 ± 1	83 ± 5	2.4 ± 0.8	176 ± 2	14.0 ± 0.6	31 ± 2
	Treated	57 ± 2	84 ± 3	1.0 ± 0.5	176 ± 1	18.9 ± 0.5	48 ± 8

## Data Availability

The raw data required to reproduce these findings cannot be shared at this time, due to technical or time limitations. The processed data required to reproduce these findings cannot be shared at this time, due to technical or time limitations.
